# Identification and Biochemical Characterization of an Acid Sphingomyelinase-Like Protein from the Bacterial Plant Pathogen *Ralstonia solanacearum* that Hydrolyzes ATP to AMP but Not Sphingomyelin to Ceramide

**DOI:** 10.1371/journal.pone.0105830

**Published:** 2014-08-21

**Authors:** Michael V. Airola, Jessica M. Tumolo, Justin Snider, Yusuf A. Hannun

**Affiliations:** 1 Department of Medicine and the Stony Brook University Cancer Center, Stony Brook University, Stony Brook, New York, United States of America; 2 Department of Biochemistry and Molecular Biology, Medical University of South Carolina, Charleston, South Carolina, United States of America; University of South Florida College of Medicine, United States of America

## Abstract

Acid sphingomyelinase (aSMase) is a human enzyme that catalyzes the hydrolysis of sphingomyelin to generate the bioactive lipid ceramide and phosphocholine. ASMase deficiency is the underlying cause of the genetic diseases Niemann-Pick Type A and B and has been implicated in the onset and progression of a number of other human diseases including cancer, depression, liver, and cardiovascular disease. ASMase is the founding member of the aSMase protein superfamily, which is a subset of the metallophosphatase (MPP) superfamily. To date, MPPs that share sequence homology with aSMase, termed aSMase-like proteins, have been annotated and presumed to function as aSMases. However, none of these aSMase-like proteins have been biochemically characterized to verify this. Here we identify RsASML, previously annotated as RSp1609: acid sphingomyelinase-like phosphodiesterase, as the first bacterial aSMase-like protein from the deadly plant pathogen *Ralstonia solanacearum* based on sequence homology with the catalytic and C-terminal domains of human aSMase. A biochemical characterization of RsASML does not support a role in sphingomyelin hydrolysis but rather finds RsASML capable of acting as an ATP diphosphohydrolase, catalyzing the hydrolysis of ATP and ADP to AMP. In addition, RsASML displays a neutral, not acidic, pH optimum and prefers Ni^2+^ or Mn^2+^, not Zn^2+^, for catalysis. This alters the expectation that all aSMase-like proteins function as acid SMases and expands the substrate possibilities of this protein superfamily to include nucleotides. Overall, we conclude that sequence homology with human aSMase is not sufficient to predict substrate specificity, pH optimum for catalysis, or metal dependence. This may have implications to the biochemically uncharacterized human aSMase paralogs, aSMase-like 3a (aSML3a) and aSML3b, which have been implicated in cancer and kidney disease, respectively, and assumed to function as aSMases.

## Introduction

Sphingomyelinases (SMases) are enzymes that catalyze the hydrolysis of sphingomyelin (SM) to generate ceramide (Cer) and phosphocholine [1]–[4]. Three families of SMases have been identified (acid, neutral, and alkaline) that are distinguished by their pH optima, protein fold, subcellular localization, primary structure, and metal dependence [1], [3], [5], [6]. Acid SMase (aSMase) was the first identified human SMase and is encoded by the SMPD1 gene [2]. ASMase is required for SM turnover in the lysosome and aSMase deficiency is the underlying cause of the genetic diseases Niemann-Pick Type A and B [2]. ASMase has also been shown to play important roles in atherosclerosis [7], cystic fibrosis [8], Wilson’s disease [9], bacterial infection [8], and apoptosis [2]. Two additional uncharacterized human proteins, aSMase-like 3a (aSML3a) and aSML3b, belong to the aSMase-protein superfamily. ASML3a and aSML3b have been implicated in cancer [10]–[12] and kidney disease [13]–[16], respectively.

**Figure 1:**
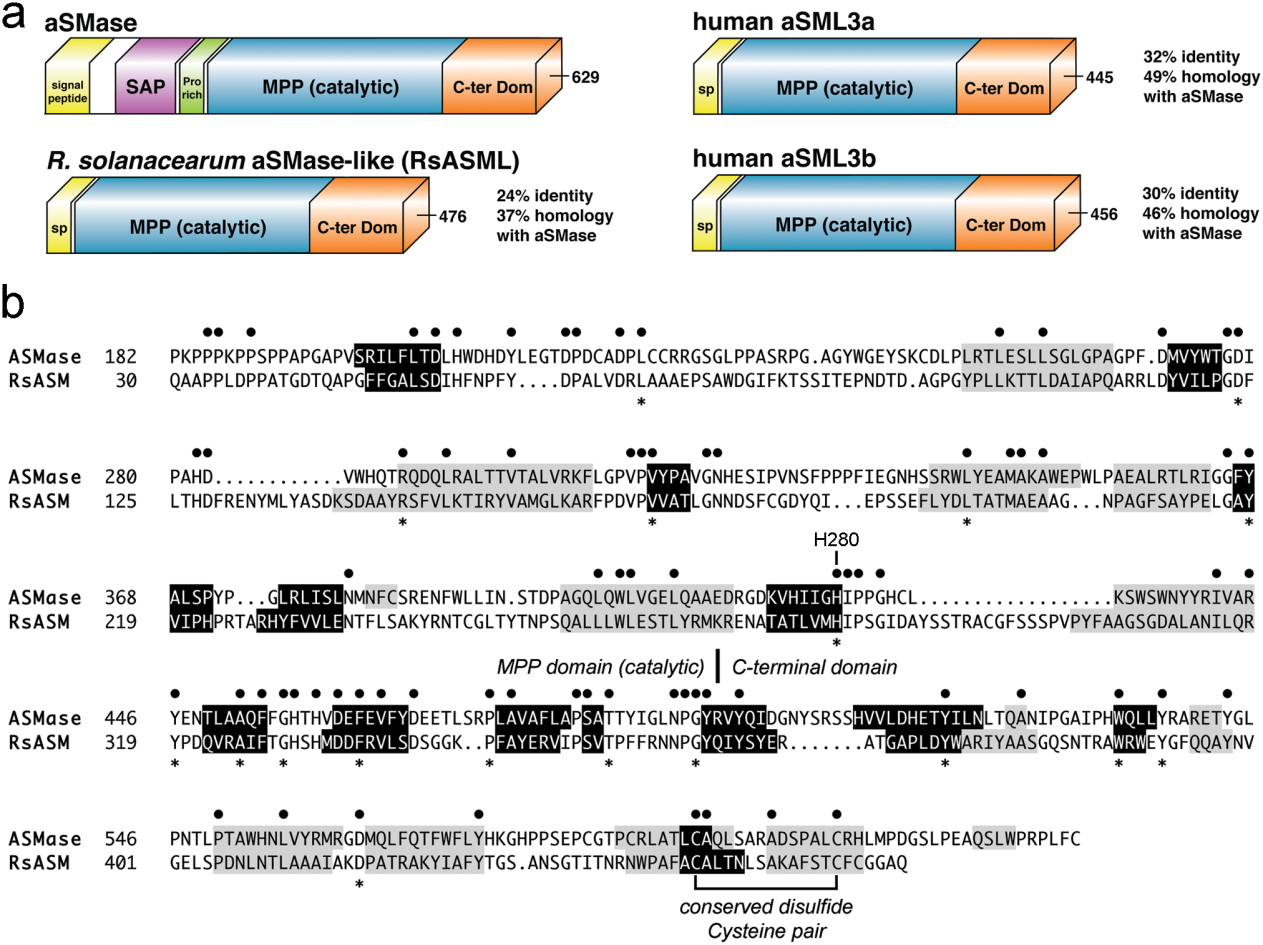
Domain architecture and sequence alignment of RsASML and human aSMase. (a) Domain architecture of acid SMase and acid-SMase-like proteins. Human aSMase contains three domains: a SAP (Sphingolipid-Activating Protein) involved in lipid binding, a MetalloPhosPhatase (MPP) catalytic domain, and a C-terminal domain required for activity but of unknown function. RsASML, from the bacteria *R. solanacearum*, and two human proteins, acid SMase-like 3a (aSML3a) and aSML3b of unknown function, share homology with the catalytic and C-terminal domains of human aSMase. (b) Sequence alignment of human aSMase and RsASML highlighting predicted secondary structure elements using Jpred3 (black = beta strands, grey = alpha helices). Black circles above denote identical residues. Asterisks below sequence indicate conserved residues identified in Niemann-Pick Type A or B patients. A known disulfide cysteine pair in human aSMase, conserved in RsASML, is noted.

The genetic and biochemical properties of aSMase have been well characterized [2]. SM hydrolysis by aSMase displays an acidic pH optima and requires Zinc for activity [2], [17]. The aSMase protein is comprised of three parts: a sphingolipid-activator protein (SAP)-like domain that aids in SM extraction from the membrane and exposure to the catalytic domain [18], a catalytic domain belonging to the metallo-phosphatase (MPP) superfamily, and a C-terminal domain of unknown structure and function (Fig. 1) [2]. A number of different inactivating mutations have been identified in Niemann-Pick Type A and B patients, which defines all three-protein domains as necessary for proper enzymatic function [2].

*Ralstonia solanacearum* is a deadly plant pathogen that causes southern bacterial wilt, infects agriculturally important crops (tomato, potato, pepper, eggplant, tobacco, banana, etc.), and has a broad geographic distribution [19], [20]. *R. solanacearum* is extremely lethal and expresses over a hundred different pathogenicity factors upon plant infection [19]–[22]. Currently, there is no method to control this pathogen and infected fields can rarely be reused, even after crop rotation with nonhost plants [23]. As such, specific strains are under quarantine status in the United States, Europe, and around the world.

Bacterial homologues of neutral SMases have been identified in many pathogens including *Bacillus cereus*, *Staphylococcus aureus, Clostridium perfringens, Listeria ivanovii* and *Streptomyces griseocarneus* [1], [3], [4], [24], [25]. In these pathogenic bacteria, the secreted neutral SMases are toxins that catalyze the hydrolysis of SM on the outer plasma membrane leaflet of erythrocytes and lymphocytes, causing hemolysis, lymphotoxicity, and septicemia [24]–[26]. Bacterial neutral SMases have served as important models for understanding the structure and biochemistry of the mammalian neutral SMase family [3]. Although bacterial neutral SMases are common, there have been no bacterial homologues identified to date for aSMase.

Here we identify and biochemically characterize the gene RSp1609, herein referred to as RsASML, as the first bacterial aSMase-like protein from the deadly plant pathogen *R. solanacearum*. We find that the RsASML protein, unlike aSMase, cannot hydrolyze SM to Cer but appears to be an ATP diphosphohydrolase, which catalyzes the hydrolysis of ATP and ADP to AMP. In addition, the biochemical properties of RsASML differ from aSMase, displaying a neutral pH optima and a Ni^2+^ metal dependence. Overall, this work broadens the substrates and pH optima of aSMase-like proteins and presents the possibility that *R. solanacearum* uses ATP hydrolysis to aid in plant pathogenicity. In addition, the identification of a bacterial aSMase-like protein suitable for X-ray crystallography studies may aid in future studies defining the structural features of the aSMase protein superfamily.

## Experimental Procedures

### Protein overexpression and purification

The RsASML gene, encompassing amino acids 30–476, which lacks a putative N-terminal secretory signal peptide (residues 1–29), from *R. solanacearum GMI1000* was PCR amplified from a cosmid (a kind gift from Stephane Genin, INRA, Toulouse, FRA). The PCR product was cloned into the *E. coli* overexpression vector ppSUMO using BamHI and NotI restriction sites. The resulting translated protein contained a cleavable N-terminal HisTag/SUMO-RsASML peptide. The RsASML ppSUMO plasmid was transformed into Origami 2 (DE3) cells for protein expression.

Cells harboring the RsASML ppSUMO plasmid were grown at 37°C in Terrific broth to an OD ∼2.0. The temperature was reduced to 15°C, and after one hour at 15°C protein production was induced with 30 mg IPTG per liter. Twenty hours after IPTG induction, cells were centrifuged and cell pellets were stored at −80°C.

Cells were resuspended in Buffer A (25 mM HEPES, pH 7.5, 500 mM NaCl, 5% glycerol, and 60 mM imidazole) and lysed by sonication. After centrifugation the supernatant was applied to a 5 mL HisTrap FF column (GE Healthcare), washed extensively with Buffer A, and eluted with Buffer B (25 mM HEPES, pH 7.5, 500 mM NaCl, 5% glycerol, and 300 mM imidazole). Fractions containing the His/SUMO-RsASML protein were collected, and the His/SUMO-tag was removed by overnight incubation with the purified SUMO-protease, ULP-1. Digested protein was applied to a Hi-Load 26–60 Superdex 200 size-exclusion column equilibrated with 10 mM HEPES, pH 8.0, 50 mM NaCl. Fractions containing the RsASML protein were pooled and concentrated to 5–10 mg/mL, aliquoted, and flash frozen.

### Para-nitrophenol (pNP)-based assays

Purified RsASML protein was incubated with the pNP-based substrates: para-nitrophenol phosphate (pNPP), para-nitrophenol phosphocholine (pNPPC, synonym: O-(4-Nitrophenylphosphoryl)choline), and pNP-thymidine 5′-monophosphate (pNP-TMP, synonym: Thymidine 5′-monophosphate p-nitrophenyl ester) in a standard 96-well plate at 25°C. A temperature of 25°C was used for activity assays as incubation at 37°C resulted in a decrease of RsASML enzyme activity over time, presumably due to protein instability. pNP formation was followed by monitoring the change in absorbance at 405****nm over time using a BioTek Synergy HT microplate reader. The extinction coefficient for pNP at 405****nm is 18,000 M^−1^cm^−1^. K_m_ and V_max_ values were determined by plotting the initial velocity (**µ**M/min) of the reaction versus the concentration of substrate and using a non-linear regression analysis. k_cat_ values (min^−1^) were determined by dividing the V_max_ values (µM/min) by the concentration of the enzyme RsASML (µM). For pH screening, multiple endpoint assays were used to calculate the initial velocity, where the reaction was quenched by addition of 1 N NaOH and the absorbance was immediately measured.

### SMase assays

Sphingomyelin, labeled with ^14^C in the phosphocholine headgroup, was incorporated in Triton X-100 micelles by sonication and incubated with RsASML or Bc-nSMase (*Bacillus cereus* nSMase) proteins at room temperature in their respective optimal reaction buffers. Reactions were quenched by addition of 1.5 mL chloroform and 400 µL water. ^14^C labeled phosphocholine was extracted using a standard Folch extraction by collecting the aqueous layer and the radioactive products were quantified using a Beckman LS6500 Scintillation Counter as described previously [27].

### NBD-lyso-SM and NBD-lyso-PC assays

0.4 mM Nitrobenzoxadiazol-lyso-sphingomyelin (NBD-lyso-SM) and Nitrobenzoxadiazol-lyso-phosphatidylcholine (NBD-lyso-PC) (Avanti Polar Lipids) stock solutions were prepared by first solubilizing in Methanol (final stock concentration = 8% v/v) and after addition of BSA (4 mg/mL). Stock solutions were stored at −20°C until use. Reactions were initiated by addition of RsASML or Bc-nSMase proteins to the NBD-lyso lipids (20 µM final) in their respective reaction buffers (RsASML: 1 mM NiCl_2_, 10 mM HEPES, pH 8.0 or Bc-nSMase: 10 mM MgCl_2_, 25 mM Tris, pH 7.5). The reaction was quenched by addition of 400 µL Chloroform/Methanol/HCl (100∶200∶1). An additional 120 µL Chloroform and 120 µL 2 M KCl were added to this solution, vortexed, and centrifuged at 3000 rpm for 10 min. 200 µL of the organic layer was removed, placed in a clean glass tube, and dried. The extracted lipids were resuspended in 50 µL of Chloroform/Methanol (1∶1) and 5 µL was spotted onto a clean TLC plate. The plate was placed in a TLC chamber equilibrated with Chloroform/Methanol/Water (65∶35∶8) to separate the reaction products. Plates were visualized using a Typhoon FLA 7000 with excitation and emission.

### ATP hydrolysis assays

RsASML was incubated with adenosine-based nucleotides (1 mM) in reaction buffer (10 mM HEPES, pH 8.0, 1 mM NiCl_2_) for 1 hr at RT. Reactions were quenched by addition of 80 µL 1 M stock of Acetic Acid/Sodium Acetate (14.7/5.3), 560 µL water, and 30 µL of chloroacetaldehyde (50% solution). The reaction products were derivatized by incubation overnight at 60°C to form the fluorescent etheno-derivatived products.

High-pressure liquid chromatography (HPLC) was performed using a Waters 1525 Binary HPLC Pump, Waters 717 plus autosampler, and Shimandzu RF-551 Spectrofluorometric detector. The derivatized reaction products were diluted 100 fold in Buffer A (0.1 M Potassium phosphate buffer, pH 6.0) and applied to a Luna 5u C18 (2) 100A column. Products were separated using a stepwise gradient against 50/50 mixture of Buffer A and Methanol. Emission and excitation values of 275 nm and 415 nm were used to detect the fluorescent products.

## Results

### Identification of a bacterial aSMase-like protein

Human aSMase is heavily glycosylated making it a difficult crystallization target [2], [28], [29]. To aid in structurally defining the aSMase protein fold, we searched for bacterial aSMase-like proteins that shared sequence homology with human aSMase and could be used for structural studies. A BLAST search [30] revealed a number of bacterial proteins that shared significant homology with the catalytic domain of human aSMase, and a few that shared homology with both the catalytic and C-terminal domains of aSMase (Table 1). None of these proteins shared additional homology with the SAP domain of aSMase.

**Table 1:** List of bacterial aSMase homologues identified by a BLAST search.

NCBI#	Bacterial organism	Max Identity	Positive Homology	Gaps
YP_002951829	*Desulfovibrio magneticus RS-1*	27% (113/411)	40% (165/411)	21% (87/411)
YP_002138293	*Geobacter bemidjiensis Bem*	24% (107/449)	35% (160/449)	22% (101/449)
ZP_06188574	*Legionella longbeachae D-4968*	26% (78/304)	40% (123/304)	19% (58/304)
ZP_01909010	*Plesiocystis pacifica SIR-1*	25% (87/346)	42% (147/346)	21% (73/346)
YP_005055725	*Granulicella mallensis MP5ACTX8*	25% (74/298)	38% (115/298)	16% (48/298)
ZP_10138266	*Fluoribacter dumoffii Tex-KL*	25% (56/228)	45% (104/228)	9% (22/228)
**YP_002257058**	***Ralstonia solanacearum GMI1000***	**24% (107/447)**	**37% (168/447)**	**23% (103/447)**

Given that the well-characterized bacterial homologues to human neutral SMase (nSMase) derive from pathogenic organisms [1], [3], [31], [32]; we reasoned that an aSMase-like protein from a bacterial pathogen was more likely to exhibit SMase activity. Given none of the bacterial proteins that we identified were from human pathogens, we selected the protein RsASML from the plant pathogen *R. solanacearum* for further biochemical analysis (Table 1). The primary sequence of RsASML shared positive homology and predicted secondary structure with human aSMase over the entire catalytic and C-terminal domains (Fig. 1b). This included a C-terminal cysteine pair known to form a disulfide in human aSMase [33], as well as 18 amino acids mutated in Niemann-Pick Type A or B patients [34]–[43]. A reverse BLAST search with the RsASML protein sequence identified RsASML as belonging to the Metallophosphatase (MPP)_aSMase protein superfamily with an E-value of 5.22e^−48^. The list of close sequence homologues to RsASML found 30 bacterial proteins sharing the highest degree of sequence homology, followed by a long list of eukaryotic homologues to aSML3a, aSML3b, and aSMase. Overall, this suggests that RsASML shares similar protein architecture to aSMase and is one of a few bacterial proteins belonging to the MPP_aSMase protein superfamily.

### Expression of RsASML requires intra-molecular disulfide formation

**Figure 2:**
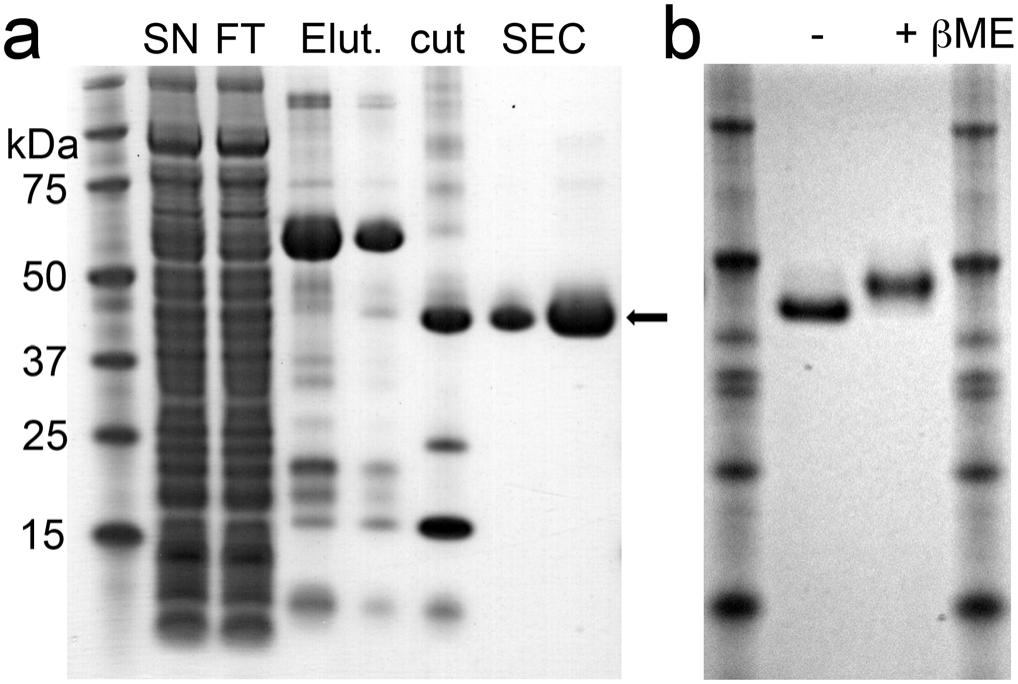
SDS-PAGE of purified RsASML protein. (a) SDS-PAGE of RsASML protein purification stained with coomassie blue. The arrow indicates the final protein purity used in biochemical assays. Abbreviations: SN: supernatant, FT: flow-through from Ni-Column, Elut: Elution from Ni-Column, Cut: Elution fractions incubated with ULP-1, a SUMO specific-protease, SEC: Size-exclusion chromatography. (b) SDS-PAGE of RsASML protein ran under non-reducing (−βME) and reducing (+βME) conditions induces a band shift, consistent with RsASML containing intra-molecular disulfide bonds.

Initial attempts to overexpress and purify RsASML from *E. coli* BL21 (DE3) cells failed. Human aSMase contains a number of intra-molecular disulfide bonds that are required for activity [33]. We hypothesized that RsASML may also require intra-molecular disulfide formation for proper folding and activity. Indeed, overexpression of a SUMO-RsASML fusion in Origami2 (DE3) cells, which promote disulfide formation in the *E. coli* cytoplasm, did result in the generation of soluble protein that could be purified to >95% homogeneity using Nickel and size-exclusion columns (Fig. 2a). SDS-PAGE samples of RsASML run under reducing and non-reducing conditions resulted in a shift in the protein band, which confirmed the presence of intra-molecular disulfides in RsASML (Fig. 2b).

### Biochemical characterization of RsASML

**Figure 3:**
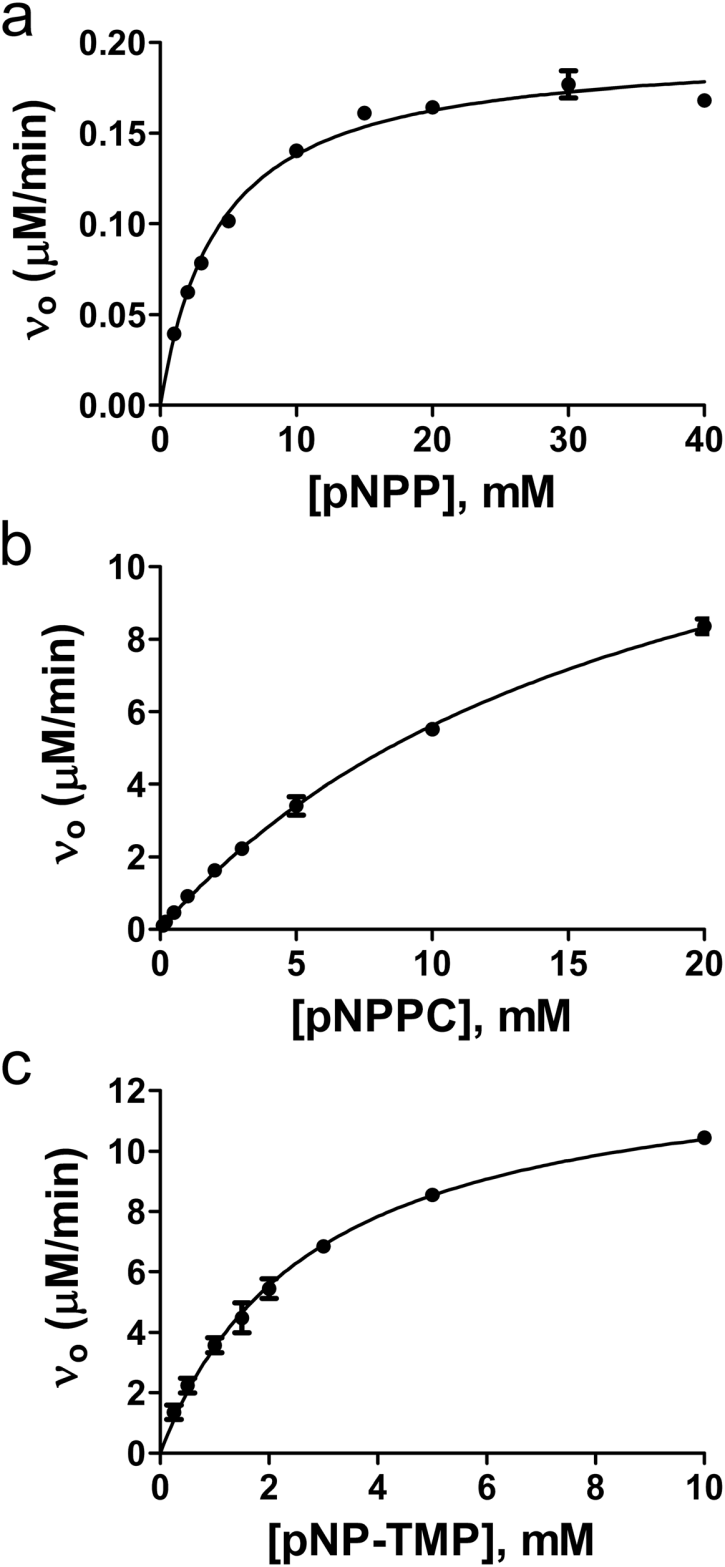
Michaelis-Menten kinetics of RsASML vs. different pNP-based substrates. Concentration dependence of RsASML activity towards (a) pNPP, (b) pNPPC, (c) pNP-TMP. All reactions were carried out in 10 mM HEPES, pH 8.0, 1 mM NiCl_2_, with 100 nM RsASML protein.

**Figure 4:**
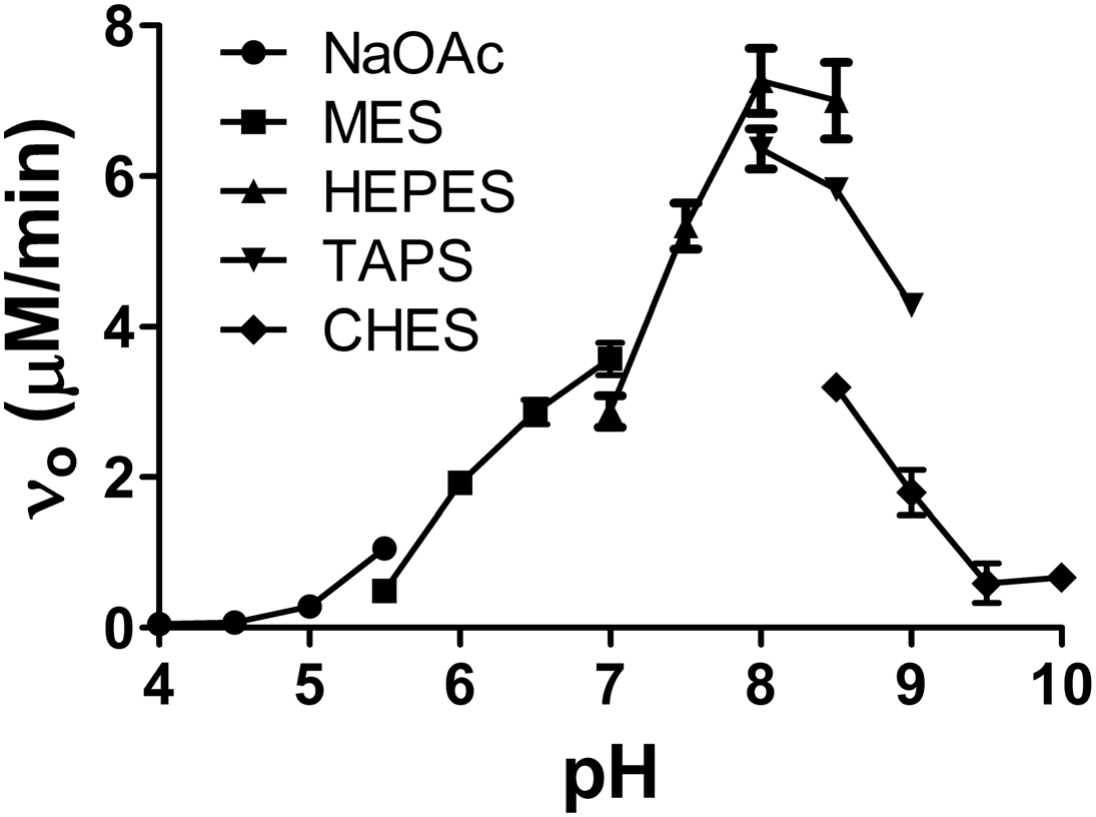
pH dependence of RsASML activity. RsASML activity towards pNPPC at different pH’s. The buffers for each pH are as indicated. Reaction conditions were 10 mM HEPES, pH 8.0, 1 mM NiCl_2_, 10 mM pNPPC with 100 nM RsASML protein.

**Figure 5:**
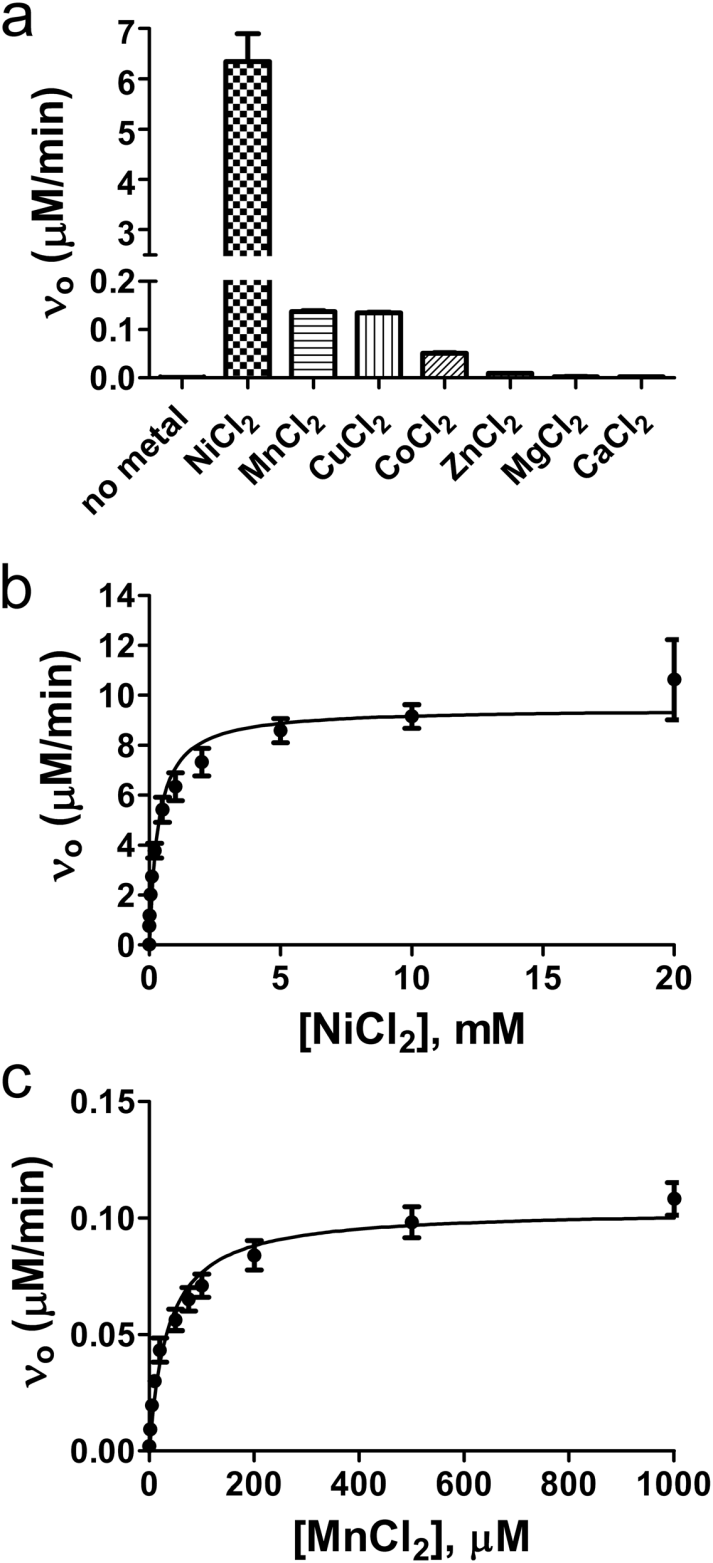
Metal dependence of RsASML activity. (a) RsASML activity towards pNPPC with different divalent metals. Reaction conditions were 10 mM HEPES, pH 8.0, 10 mM pNPPC with 100 nM RsASML protein at a concentration of 1 mM for each metal. (b) Dependence of RsASML activity on Ni^2+^ concentration. (c) Dependence on Mn^2+^ concentration.

To characterize the biochemical properties of RsASML we started by testing the ability of RsASML to hydrolyze generic phosphatase substrates belonging to the para-nitrophenol (pNP) family. RsASML was able to catalyze the hydrolysis of phosphate from para-nitrophenol phosphate (pNPP) (Fig. 3a), as well as a phosphocholine headgroup from para-nitrophenol phosphocholine (pNPPC) (Fig. 3b). Significantly higher activity was detected towards the soluble SM-mimic pNPPC, which shares the phosphocholine headgroup with SM (Fig. 3b). After confirming linearity of the reaction versus time and protein concentration, we determined that RsASML, unlike human aSMase, displayed activity over a broad range between pH 6–9, with maximal activity at the neutral pH of 8 (Fig. 4). RsASML activity was metal dependent with Ni^2+^ stimulating activity far above all other metals (Fig. 5). Significant activity over baseline was also detected for the metals Mn^2+^>Cu^2+^>Co^2+^. Ni^2+^ displayed a relatively high K_metal_ of 336 µM and a V_max_ of 9.5 µM/min (Fig. 5b, Table 2). In comparison, Mn^2+^ had a lower K_metal_ of 35.4 µM but an ∼100 fold lower V_max_ of 0.1 µM/min (Fig. 5c, Table 2). Notably, Zn^2+^, which human aSMase utilizes for SM hydrolysis, provided the weakest activity of the transition metals tested (Fig. 5a).

**Table 2:** K_metal_ and V_max_ values for NiCl_2_ and MnCl_2_.

	NiCl_2_	MnCl_2_
V_max_(µM/min)	9.47+/−0.38	0.104+/−0.004
K_metal_ (µM)	336.2+/−62.64	35.35+/−5.29
V_max_/K_metal_	0.0282	0.0029

In addition to pNPP and pNPPC, RsASML could also hydrolyze the substrate para-nitrophenol-thymidine 5′-monophosphate (pNP-TMP) (Fig. 3c), which requires nucleotide phosphodiesterase activity for hydrolysis. Interestingly, RsASML displayed higher activity towards pNP-TMP versus the SM-mimic pNPPC under identical conditions (Fig. 3). A comparison of the k_cat_ and K_m_ values found that RsASML had similar k_cat_ values for pNPPC and pNP-TMP (Fig. 3, Table 3). The increase in activity was due to a higher affinity of RsASML towards pNP-TMP versus pNPPC, with a K_m_ approximately 10 fold lower (Table 3).

**Table 3:** Michaelis-Menten values for pNP-based substrates.

	pNPP	pNPPC	pNP-TMP
k_cat_ (min^−1^)	2.01+/−0.04	163.67+/−7.46	135.52+/−4.65
K_m_ (mM)	4.29+/−0.27	18.55+/−1.41	2.79+/−0.22
k_cat_/K_m_	0.47	8.82	48.64

**Figure 6:**
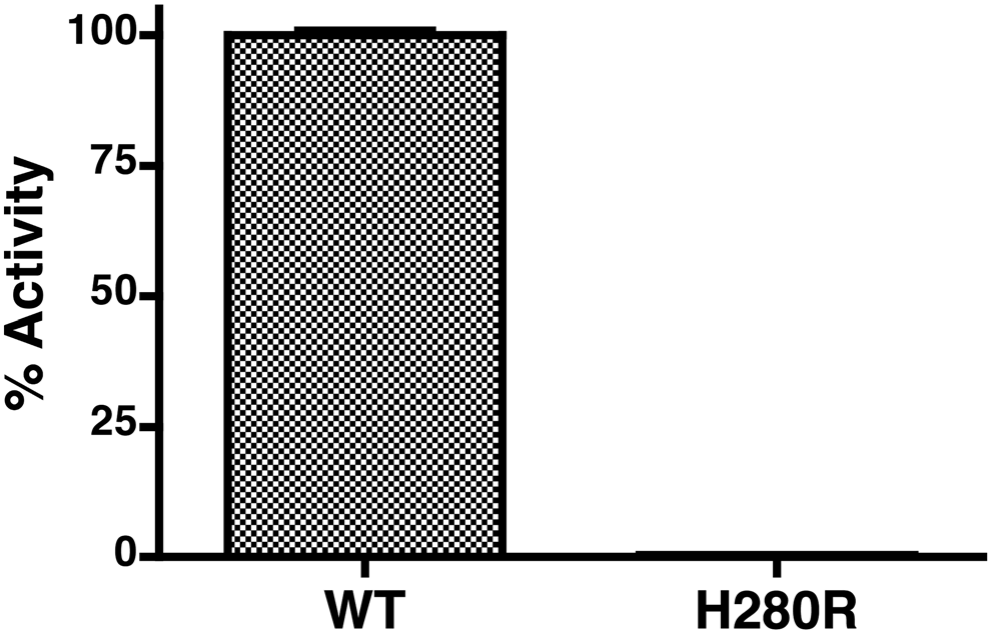
A corresponding Niemann-Pick mutation inactivates RsASML. RsASML activity of WT and H280R proteins towards pNPPC. The H280R substitution in RsASML corresponds to the H427R mutation in aSMase found in a subset of Niemann-Pick Type A patients.

To ensure that the activity we were observing was due to RsASML and not any minor contaminating *E. coli* proteins, we generated RsASML H280R by substituting Histidine 280 to Arginine using site-directed mutagenesis. This mutation in RsASML corresponds to the H427R mutation in human aSMase found in a subset of Niemann-Pick Type A patients [37]. RsASML H280R did not display any activity towards pNPPC (Fig. 6) confirming the hydrolysis was due to the presence of RsASML.

### RsASML does not hydrolyze sphingomyelin or other phosphocholine-based lipids

**Figure 7:**
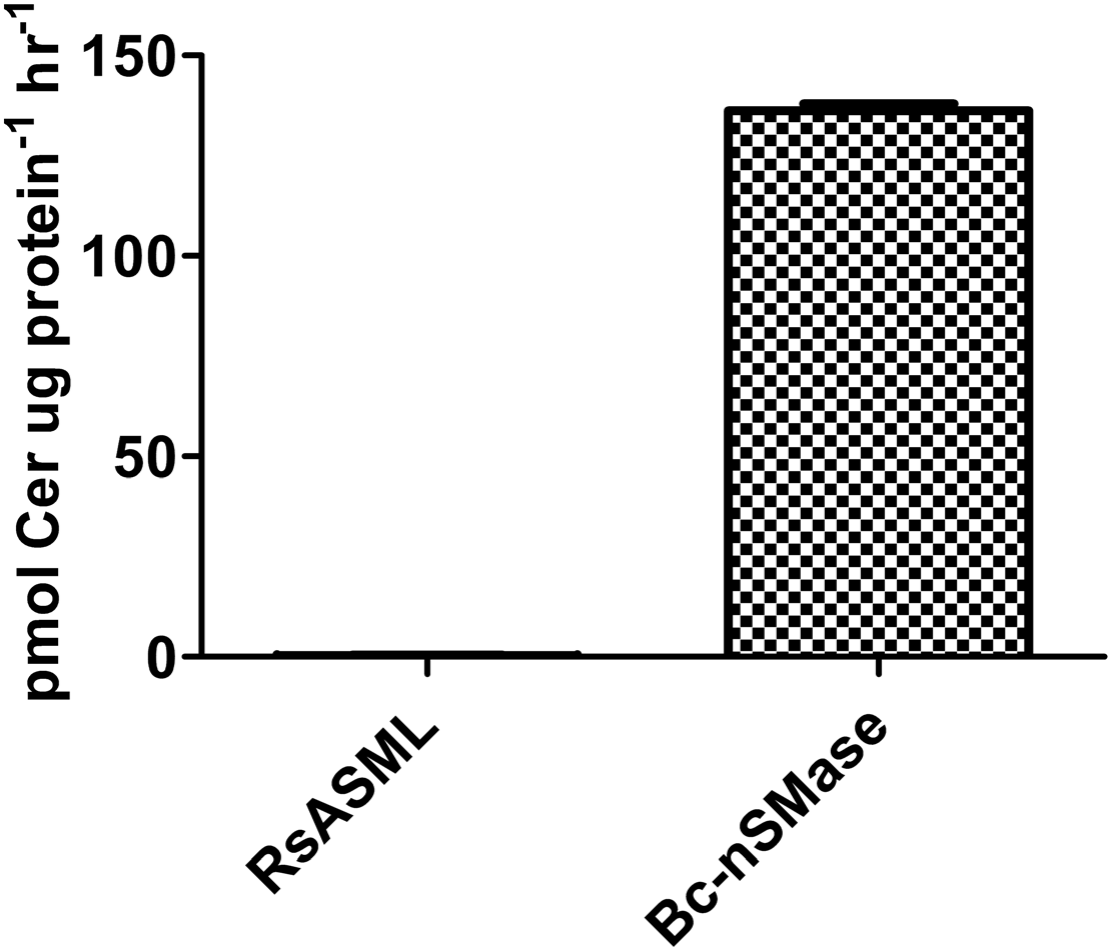
Sphingomyelinase activity of RsASML and Bc-nSMase. RsASML reaction conditions: 10 mM HEPES, pH 8.0, 1 mM NiCl_2_. Bc-nSMase (*Bacillus cereus* nSMase) reactions conditions: 10 mM HEPES, pH 7.5, 10 mM MgCl_2_.

**Figure 8:**
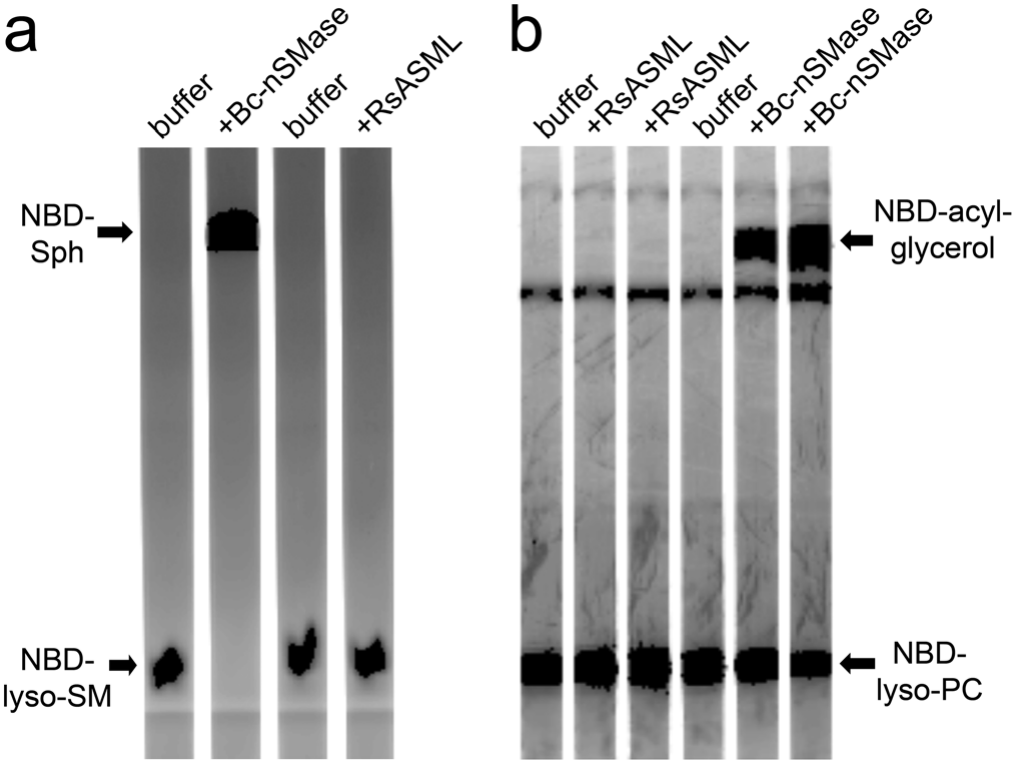
Phospholipase activity of RsASML and Bc-nSMase towards NBD-lyso-SM and NBD-lyso-PC. (a) Fluorescently imaged TLC plate of NBD-lyso-SM reactions. (b) Fluorescently imaged TLC plate of NBD-lyso-PC reactions. RsASML reaction conditions: 10 mM HEPES, pH 8.0, 1 mM NiCl_2_. Bc-nSMase (*Bacillus cereus* nSMase) reactions conditions: 10 mM HEPES, pH 7.5, 10 mM MgCl_2_.

Having established optimal reaction conditions; we assessed the ability of RsASML to hydrolyze SM and other lipid substrates containing a phosphocholine headgroup. Using a standard SMase assay where SM is incorporated into Triton-X 100 mixed micelles, we found that RsASML did not catalyze SM hydrolysis (Fig. 7). For comparison, we used bacterial nSMase from *B. cereus* (Bc-nSMase) as a positive control. RsASML lacks the SAP domain found in human aSMase that is thought to aid in SM extraction from the membrane and facilitate SM availability to the catalytic domain. To ensure the lack of SMase activity of RsASML was not due to the absence of a SAP domain and lack of SM access to the catalytic domain, we assessed the activity of RsASML in more soluble lipid systems. These systems utilized the fluorescently labeled lipids NBD-lyso-SM and NBD-lyso-phosphatidylcholine (NBD-lyso-PC) complexed with fatty acid-free BSA. RsASML did not hydrolyze NBD-lyso-SM and NBD-lyso-PC, while Bc*-*nSMase hydrolyzed both (Fig. 8). From these experiments, we concluded that RsASML is unlikely to be a SMase and may catalyze the hydrolysis of a different substrate.

### Competitive Inhibition Assay to Identify High Affinity Substrates

**Figure 9:**
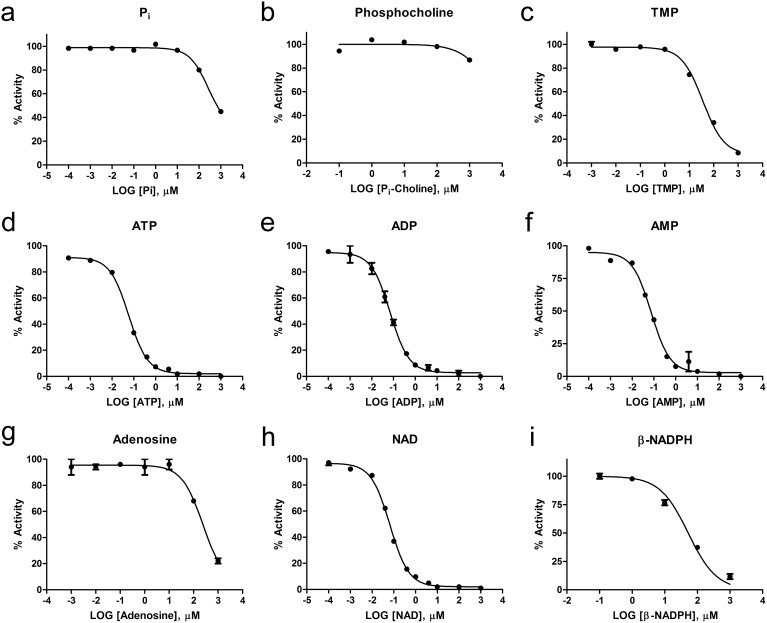
Competitive inhibition of RsASML activity by small molecules. RsASML activity towards pNPPC was assessed in the presence of varying concentrations of phospho-containing small molecules. Data points were fit using a nonlinear regression (log [inhibitor] vs. normalized response) in the program PRISM. All reactions were carried out in 10 mM HEPES, pH 8.0, 1 mM NiCl_2_ with 61.3 nM RsASML protein and 5 mM pNPPC.

To aid in the identification of high affinity substrates, we used a competitive inhibition assay. The phosphatase activity of RsASML towards pNPPC was assessed in the presence of varying concentrations of phospho-containing small molecules. Lower IC_50_ values indicate more potent inhibition. As an initial verification, we used the corresponding phospho-moieties from the set of pNP-based substrates. Consistent with the measured affinities of RsASML towards the pNP-based substrates, TMP displayed the lowest IC_50_ compared to free phosphate and phosphocholine (Fig. 9, Table 4). Other small molecules, representing protein-phosphatase, poly-phosphatase, and phospholipase activities, yielded relatively high IC_50_ values (Table 4).

**Table 4:** IC_50_ values for inhibition of RsASML pNPPC activity.

Molecule	IC_50_ (µM)
Adenosine	243.2
ADP	0.07049
AMP	0.07499
ATP	0.05864
β-NADPH	52.15
cAMP	63.72
cGMP	32028
CTP	1502
GTP	44.21
L-α-glycerolphosphate	745.9
L-α-glycerolphosphocholine	64.61
NAD^+^	0.06632
Phosphate	265
Phospho-Threonine	6671
Phosphocholine	4430
Pyro-Phosphate	638
TMP	35.85
TTP	200.8
UTP	1362

IC_50_ values were determined using a nonlinear regression (log [inhibitor] vs. normalized response) in the program PRISM. All reactions were carried out in 10 mM HEPES, pH 8.0, 1 mM NiCl_2_ with 61.3 nM RsASML protein and 5 mM pNPPC.

### Adenosine-based nucleotides are potent inhibitors of RsASML

Given that RsASML displayed nucleotide phosphodiesterase activity towards pNP-TMP, we assessed the IC_50_’s of different nucleotides. ATP sharply inhibited RsASML activity towards pNPPC, while other tri-phosphate-nucleotides did not (Fig. 9, Table 4). A comparison of the IC_50_’s of adenosine-based nucleotides found that ATP, ADP, and AMP all potently inhibited RsASML activity in the nanomolar range. Notably, the concentration of RsASML protein was nearly equivalent to the IC_50_ concentrations of ATP, ADP, and AMP. Adenosine, which has no phosphate group, inhibited weakly with a significantly higher IC_50_. NAD^+^, which contains a di-phosphate adenosine moiety, had a comparable IC_50_ to ATP/ADP/AMP. In contrast, additions or modifications of the AMP backbone resulted in diminished inhibition as seen for β-NADPH and cyclic-AMP (cAMP) (Fig. 9, Table 4).

### RsASML can hydrolyze ATP and ADP to AMP

**Figure 10:**
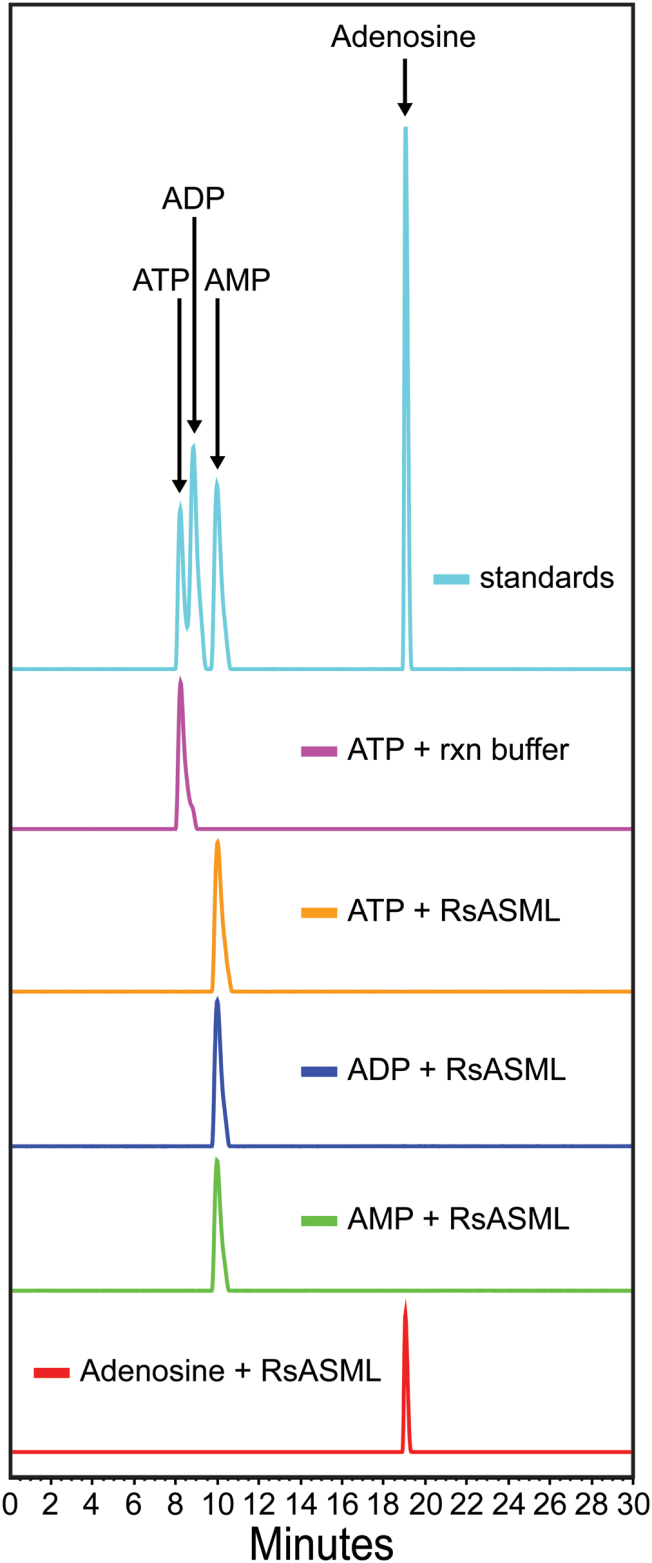
RsASML catalyzes the hydrolysis of ATP and ADP to AMP. HPLC chromatograms of adenosine-based nucleotides incubated with reaction buffer or RsASML protein. All reactions were carried out in 10 mM HEPES, pH 8.0, 1 mM NiCl_2_ with 1 µM RsASML protein.

The potent inhibition of ATP, ADP, and AMP suggested that RsASML might be a nucleotide phosphodiesterase specific for adenosine-based nucleotides. The ability of RsASML to hydrolyze adenosine-based nucleotides was directly assessed. RsASML catalyzed the hydrolysis of both ATP and ADP to form AMP (Fig. 10). AMP was not further hydrolyzed to adenosine. Overall, this suggests that RsASML is an ATP diphosphohydrolase.

## Discussion

Here we identify and biochemically characterize RsASML, the first bacterial aSMase-like protein, as well as the first aSMase-like protein to be biochemically characterized. Although RsASML shares significant sequence homology with the catalytic and C-terminal domains of human aSMase, the protein does not display SMase activity. Rather, our in vitro characterization suggests RsASML may function as an ATP diphosphohydrolase. In addition to broadening the potential substrates of the MPP_aSMase protein superfamily, we find that RsASML displays a different pH optima and metal dependence to human aSMase. We note that the Ni^2+^ metal dependence is not likely to be biologically relevant as free Ni^2+^ concentrations are very low. It is more likely that Mn^2+^ is the physiological relevant metal for RsASML activity.

This work has a number of implications to the MPP_aSMase protein superfamily. To date, all aSMase-like proteins have been assumed to exhibit aSMase activity based simply on the substrate specificity of aSMase. This includes the uncharacterized human proteins aSML3a and aSML3b, which similar to RsASML also lack the SAP domain found in aSMase. This is important as aSML3a (gene name: SMPDL3A) and aSML3b (gene name: SMPDL3B) have recently been identified as necessary for cell division [44] and a potential target for treatment of the common kidney disease Focal Segmental Glomerulosclerosis (FSGS) [45], respectively. Notably, ceramide has been assumed to be the resultant bioactive product of aSML3a and aSML3b activity in these cases and others [10], [11], [14], [44]–[47]. Based on the characterization of RsASML, the MPP_aSMase protein superfamily may catalyze the hydrolysis of a variety of substrates, similar to the ectonucleotide pyrophosphatase/phosphodiesterase (ENPP) protein superfamily, whose well-characterized members ENPP1, ENPP2 (more commonly referred to as Autotaxin), and ENPP7 (alkaline SMase) catalyze the hydrolysis of ATP to AMP, lyso-PC to lyso-PA, and SM to Cer, respectively [5], [48]–[51]. That aSMase-like proteins may hydrolyze a variety of phosphate moieties is consistent with aSMase belonging to the general metallophosphatase protein superfamily, whose members include protein phosphatases and exonucleases. Therefore it is of utmost importance to define the substrate specificities of both aSML3a and aSML3b, which may include SM, ATP, or other phospho-containing molecules, to help guide future studies.

These results also raise the possibility that the substrates of S-SMase, the secreted form of aSMase, which is located in the neutral pH environment of the extracellular space and where Zinc concentrations are very low, may not be limited to SM. How S-SMase functions outside its normal acidic pH optimum is a major question that still needs to be addressed. An interesting study found that S-SMase was capable of hydrolyzing artherogenic, modified LDL-bound SM at neutral pH but not normal LDL-bound SM [52]. The work presented here, raises the distinct possibility that S-SMase may be capable of hydrolyzing additional substrates, beyond artherogenic LDL-SM [52], in the neutral pH environment of the extracellular matrix.

Furthermore, it would be interesting to evaluate the role of RsASML as a putative ATP diphosphohydrolase in the pathogenesis of *R. solanacearum* in vivo. Recently, DORN1 was identified as the long sought after plant extracellular ATP receptor [53]. DORN1 is unique in that it shares no homology with the human extracellular ATP receptors, P2X and P2Y. Extracellular ATP was found to up-regulate a highly similar set of genes that correlate with early plant wounding [53]. Concentrations of extracellular ATP, as high as 40 µM, have been measured at sites of plant physical wounding [54]. Overall, this suggests that extracellular ATP may serve as a danger signal in plants in response to physical wounding [53]. Although future work is needed to verify a role for RsASML in extracellular ATP hydrolysis during plant infection, RsASML does contain a putative secretory signal peptide. Based on this, we hypothesize that RsASML may function to lower the concentration of an extracellular ATP danger signal during plant infection and thereby aid in the pathogenesis of *R. solanacearum.*
